# The Hahnemannian’s Analysis Sheet

**Published:** 1891-02

**Authors:** 


THE HAHNEMANNIAN'S ANALYSIS SHEET. By M. A. A. Wolff, M. D., Gainesville, Texas. Sample sheets five cents each. Twenty-five sheets for one dollar.

These sheets are for the convenience of the practitioner in studying a case. The sheet is divided into a number of blank spaces, in each one of which is printed the name of a remedy. There is space after the name for writing. The idea is that in using a repertory every remedy mentioned under a particular rubric shall have a mark set opposite to it. Then studying every symptom in the same way, we shall be able to see at a glance what remedies have all the symptoms and what have not. It is an ingenious idea, and is a modification of that suggested by Dr. Edmund J. Lee, and of the later device of Dr. Alfred Heath, also of Dr. Wm. Jefferson Guernsey's ingenious arrangement of Bœnninghausen's Therapeutic Pocket Book. Every physician who makes strictly homœopathic prescriptions ought to have it. W. M. J.

